# Blood-borne phagocytes internalize urate microaggregates and prevent intravascular NETosis by urate crystals

**DOI:** 10.1038/srep38229

**Published:** 2016-12-05

**Authors:** Elmar Pieterse, Ivica Jeremic, Christine Czegley, Daniela Weidner, Mona H.C. Biermann, Susan Veissi, Christian Maueröder, Christine Schauer, Rostyslav Bilyy, Tetiana Dumych, Markus Hoffmann, Luis E. Munoz, Anders A. Bengtsson, Georg Schett, Johan van der Vlag, Martin Herrmann

**Affiliations:** 1 Department of Nephrology, Radboud University Medical Center, Nijmegen, the Netherlands; 2 Department of Internal Medicine 3, Friedrich-Alexander-University Erlangen-Nuremberg, Erlangen, Germany; 3 Institute of Rheumatology, Resavska 69, Belgrade, Serbia; 4 Danylo Halytsky Lviv National Medical University, Lviv, Ukraine; 5 Department of Rheumatology, University Hospital of Lund, Lund, Sweden

## Abstract

Hyperuricemia is strongly linked to cardiovascular complications including atherosclerosis and thrombosis. In individuals with hyperuricemia, needle-shaped monosodium urate crystals (nsMSU) frequently form within joints or urine, giving rise to gouty arthritis or renal calculi, respectively. These nsMSU are potent instigators of neutrophil extracellular trap (NET) formation. Little is known on the mechanism(s) that prevent nsMSU formation within hyperuricemic blood, which would potentially cause detrimental consequences for the host. Here, we report that complement proteins and fetuins facilitate the continuous clearance by blood-borne phagocytes and resident macrophages of small urate microaggregates (UMA; <1 μm in size) that initially form in hyperuricemic blood. If this clearance fails, UMA exhibit bipolar growth to form typical full-sized nsMSU with a size up to 100 μm. In contrast to UMA, nsMSU stimulated neutrophils to release NETs. Under conditions of flow, nsMSU and NETs formed densely packed DNase I-resistant tophus-like structures with a high obstructive potential, highlighting the importance of an adequate and rapid removal of UMA from the circulation. Under pathological conditions, intravascularly formed nsMSU may hold the key to the incompletely understood association between NET-driven cardiovascular disease and hyperuricemia.

Hyperuricemia (uric acid blood levels ≥6 (women) or 6.8 (men) mg/dL) is the major risk factor for the development of gout^1^,^2^. When the concentration of uric acid exceeds its solubility limit, monosodium urate crystals (cMSU) may form. Such cMSU initially appear as round-shaped bifringent urate microaggregates (UMA; ≤1 μm in size), which can subsequently grow in a bipolar manner to form needle-shaped monosodium urate crystals (nsMSU; up to 100 μm in size)^3^. In gout, arthritis occurs due to the deposition of nsMSU in the peripheral joints. These nsMSU may also develop within the urine, giving rise to renal calculi. The inflammatory response towards nsMSU is in part characterized by neutrophils that undergo oxidative burst and release their chromatin as neutrophil extracellular traps (NETs) during a process known as NETosis^4^,^5^,^6^,^7^. NETs are found in the synovial fluids and tissue of patients with gout, especially in those with gouty flares or granuloma formation^4^,^8^. At high neutrophil densities, NETs and nsMSU can form densely packed aggregates, that appear as tophus-like structures^9^,^10^.

In contrast to the synovial fluids or urine of hyperuricemic patients, the presence of nsMSU has never been described in blood. The presence of nsMSU in blood could have detrimental consequences for the host, since the intravascular formation of nsMSU and the consecutive induction of NETosis bear the risk of life-threatening atherosclerosis, increased vascular permeability, and thrombosis^11^,^12^,^13^.

In the present study, we investigated the crystallization of uric acid in hyperuricemic blood. We elucidated a novel mechanism by which blood-borne phagocytes remove small UMA initially formed in hyperuricemic blood before these can actually develop into NET-inducing nsMSU. If this mechanism fails, nsMSU and NETs form DNase I-resistant tophus-like aggregates with a high obstructive potential.

## Results

### Uric acid crystallizes in hyperuricemic serum and whole blood *ex vivo*

**Figure 1:**
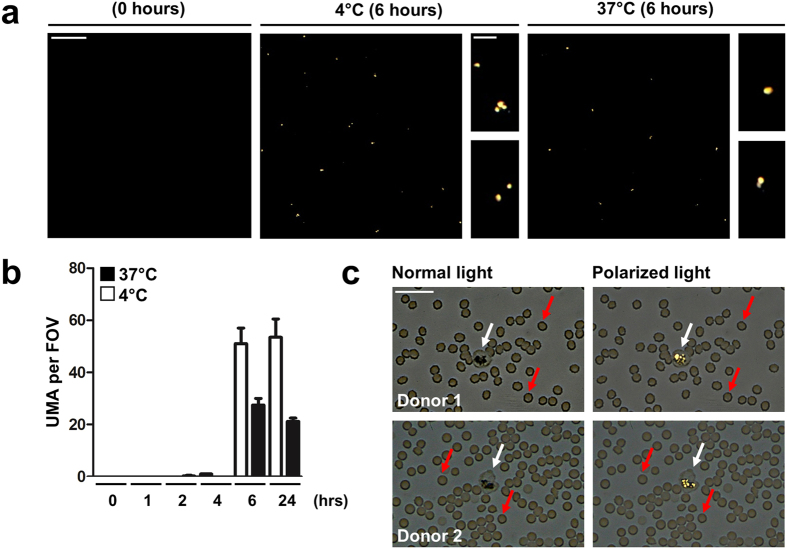
Uric acid crystallizes in hyperuricemic serum and whole blood *ex vivo*. (**a**) Small, round-shaped urate microaggregates (UMA) developed after *in vitro* incubation of hyperuricemic serum. Representative images are shown. (**b**) Crystallization of uric acid preferentially occurred at colder temperatures and was saturated after 6 hours of incubation of hyperuricemic serum. The amount of UMA was scored by polarized light microscopy whereby the number of UMA per field of view (FOV; 200x magnification) was scored. (**c**) Incubation of hyperuricemic whole blood for 6 hours *ex vivo* showed intracellular presence of UMA within blood-borne phagocytes (white arrows) in blood smears. Red arrows represent erythrocytes. Representative images are shown from two hyperuricemic gout patients. Scale bars: 20 μm (inserts: 5 μm).

To assess whether high uric acid levels can promote crystal formation in serum or whole blood, we analyzed samples from eight hyperuricemic patients and normouricemic normal healthy donors *ex vivo*. Uric acid levels in hyperuricemic patients were above 12 mg/dL, thereby exceeding the theoretical threshold of solubility for uric acid. Serum and whole blood was incubated at 4 °C or 37 °C and analyzed for crystalline structures at set time points using polarized light microscopy. After 6 hours of incubation, round-shaped UMA were observed in the sera of hyperuricemic patients (Fig. 1a) but not in those from healthy donors (not shown). Prolonged incubation (up to 24 hours) did not further increase UMA formation in hyperuricemic serum (Fig. 1b). Furthermore, healthy donor sera remained negative for UMA after *ex vivo* incubation up to 72 hours (not shown). In whole blood smears, we also detected UMA after 6 hours of incubation *ex vivo*. However, UMA were exclusively present within blood-borne phagocytes (Fig. 1c). Based on these observations, we hypothesized that blood-borne phagocytes clear UMA from hyperuricemic blood, thereby preventing intravascular formation of nsMSU and the consecutive induction of NETosis.

### Blood-borne phagocytes rapidly internalize UMA

**Figure 2:**
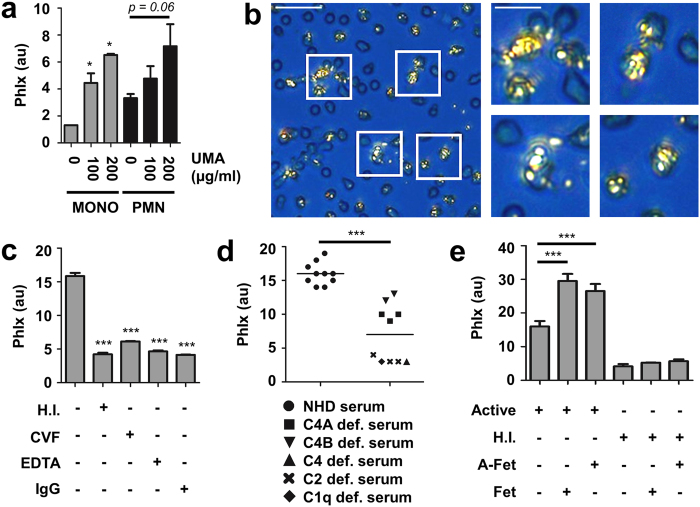
Blood-borne phagocytes rapidly internalize UMA. (**a**) Whole blood was incubated with 100 or 200 μg/ml UMA. Then their uptake by CD14+ monocytes (‘mono’) or CD16+ neutrophils (‘PMN’) was assessed by flow cytometry. (**b**) Purified neutrophils contained UMA as soon as 15 min after incubation. (**c**) Purified neutrophils ingested significantly more UMA in the presence of fresh serum containing active complement (left bar), whereas heat inactivation (H.I; 30 min, 56 °C) of serum or treatment of active serum with cobra venom factor (CVF; 20U/ml), EDTA (500 μM) and heat-aggregated IgG (1 mg/ml) prevents UMA ingestion. (**d**) Uptake of UMA by purified neutrophils was impaired in the presence of complement deficient sera (C4A, n = 3; C4B, n = 2; C4, n = 1; C2, n = 3; C1q, n = 1), when compared to sera from normal healthy donors (NHD, n = 10). (**e**) Spiking active serum with fetuin (1 mg/ml) or its enzymatically desialyated form asialofetuin (1 mg/ml) boosts the phagocytosis of UMA by neutrophils. Scale bars: 20 μm (inserts: 10 μm). ***p < 0.001, **p < 0.01, *p < 0.05, when compared to control situation where not indicated. The phagocytic indices (PhIx) were calculated by multiplying the percentage of cells with increased side scatter by the mean side scatter value.

To determine whether blood-borne phagocytes internalize UMA, we incubated healthy donor whole blood with *in vitro* generated UMA and evaluated their uptake by blood-borne phagocytes by flow cytometry and polarized light microscopy. In response to UMA, CD14^+^ monocytes dose-dependently displayed an increased side scatter when compared to untreated cells (Fig. 2a). A similar result was observed for CD16^+^ neutrophils. To confirm that the increased side scatter represented UMA internalization, we exposed neutrophils to UMA in the presence of pHrodo, which is a dye that becomes co-ingested during phagocytosis and which becomes fluorescent in the acidic milieu of the phagosome. Indeed, neutrophils with an increased side scatter were positive for pHrodo, in contrast to neutrophils with a normal side scatter, thereby confirming uptake of UMA (Supplementary Figure 1a,b and c). Importantly, the phagocytic index (PhIx) of cells exposed to UMA, as calculated by multiplying the percentage of cells with increased side scatter with the mean side scatter value, directly correlated to the mean fluorescence intensity of pHrodo (Supplementary Figure 1d). Finally, the uptake of UMA by neutrophils was recorded using polarized light microscopy (Fig. 2b; Movie S1).

### Complement proteins and fetuins facilitate phagocytosis of UMA

It has been previously reported that complement can be activated by nsMSU resulting in opsonization^14^,^15^,^16^,^17^. To assess a possible role for complement proteins in the phagocytosis of UMA, we stimulated purified neutrophils with UMA in the presence of (I) 10% active autologous serum, (II) 10% heat-inactivated serum, (III) 10% autologous Ca^2+^-depleted serum by EDTA (500 μM) or 10% autologous serum supplemented with either (IV) heat-aggregated IgG (1 mg/mL) or (V) cobra venom factor (20 U/ml). The two latter treatments (IV, V) cause complement consumption and depletion. Thus, only the fresh autologous serum (I) contained active complement and supported the uptake of UMA into purified neutrophils (Fig. 2c, Supplementary Figure 1e). Heat-aggregated IgG and EDTA are broad-spectrum inhibitors of the complement system, whereas cobra venom factor specifically inhibits the alternative pathway due to C3 consumption. To more specifically investigate the role of the classical complement pathway in UMA phagocytosis, we performed these assays in 10% serum derived from patients deficient in complement factors C4A, C4B, C4, C2 and C1q. All complement-deficient patients showed an impaired/abrogated uptake of UMA (Fig. 2d).

Fetuins are carrier proteins that bind and solubilize crystal germs from calcium phosphate^18^. Therefore, we tested whether fetuins also facilitated the phagocytosis of UMA. We observed an increase of 40% in the phagocytosis of UMA by neutrophils when 10% active autologous serum was supplemented with 1 mg/mL fetuin or its desialyated form asialofetuin (Fig. 2e). Interestingly, employing 10% heat-inactivated autologous serum, neither asialofetuin nor fetuin facilitated the phagocytosis of UMA by neutrophils. Taken together, our data reveal a cooperative mechanism between fetuins and complement proteins in facilitating the phagocytosis of UMA by neutrophils.

### Macrophages recognize and clear UMA-loaded phagocytes

**Figure 3:**
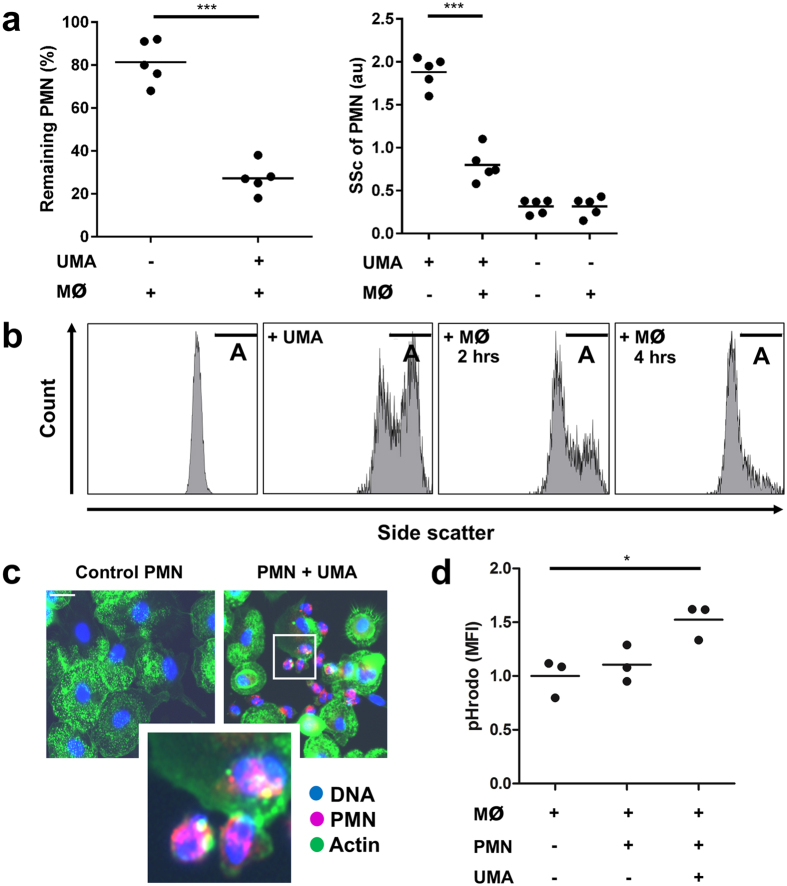
Monocyte-derived macrophages recognize and engulf UMA-containing neutrophils. (**a**) After co-incubation with macrophages for 4 hours, the amount of UMA-stimulated neutrophils, normalized to neutrophils before co-culture, significantly declined in culture supernatants (left panel). Particularly the percentage of neutrophils with an increased side scatter decreased in these co-cultures (right panel). (**b**) Representative flow cytometry charts showing that macrophages selectively recognize neutrophils with an increased side scatter (gate A). (**c**) PKH26-labeled neutrophils (red; PMN) are either attached to or taken up by monocyte-derived macrophages (green), where unstimulated neutrophils (control) are not. (**d**) pHrodo-labeled neutrophils pre-loaded with UMA are engulfed by macrophages, whereas neutrophils not exposed to UMA are not. Scale bars: 20 μm. ***p < 0.001, *p < 0.05, when compared to control situation where not indicated.

Macrophages and neutrophils share cooperative effector activities, of which one modality comprises the ability of macrophages to internalize neutrophils that express “eat-me” signals^19^. We hypothesized that the ingestion of UMA by blood-borne phagocytes results in the clearance of these cells by splenic and/or hepatic macrophages. To test this, we incubated purified neutrophils with UMA and performed co-culture experiments with monocyte-derived macrophages. After 4 hours of co-culture with macrophages, the amount of UMA-loaded neutrophils was significantly reduced, whereas naive neutrophil counts remained stable (Fig. 3a, left panel). Notably, in particular the percentage of neutrophils with an increased side scatter decreased in these co-cultures (Fig. 3a, right panel; Fig. 3b). In line with this, we confirmed by immunofluorescence microscopy that neutrophils were observed within or attached to macrophages when these neutrophils had been loaded with UMA (Fig. 3c). The uptake of UMA-loaded neutrophils by macrophages was further confirmed by using neutrophils pre-labeled with pHrodo. A pHrodo-derived fluorescent signal was obtained after co-culturing macrophages with neutrophils pre-loaded with UMA, in contrast to neutrophils that were not exposed to UMA (Fig. 3d). Finally, to assess how macrophages may recognize UMA-loaded neutrophils, we analyzed the expression of phosphatidyl serine (PS; expressed on apoptotic cells), CD11a/CD18 (LFA-1) and CD31 on neutrophils before and after exposure to UMA. Notably, it has previously been shown that these cell surface markers facilitate interactions between neutrophils and macrophages^20^,^21^,^22^. We observed that UMA-loaded neutrophils remained viable, i.e. did not expose PS, and did not alter LFA-1 expression, whereas CD31 expression was diminished (Supplementary Figure 2). In conclusion, macrophages recognize and engulf neutrophils loaded with UMA, which is possibly facilitated by the loss of CD31 expression.

### Impaired UMA clearance promotes nsMSU-induced NET release

**Figure 4:**
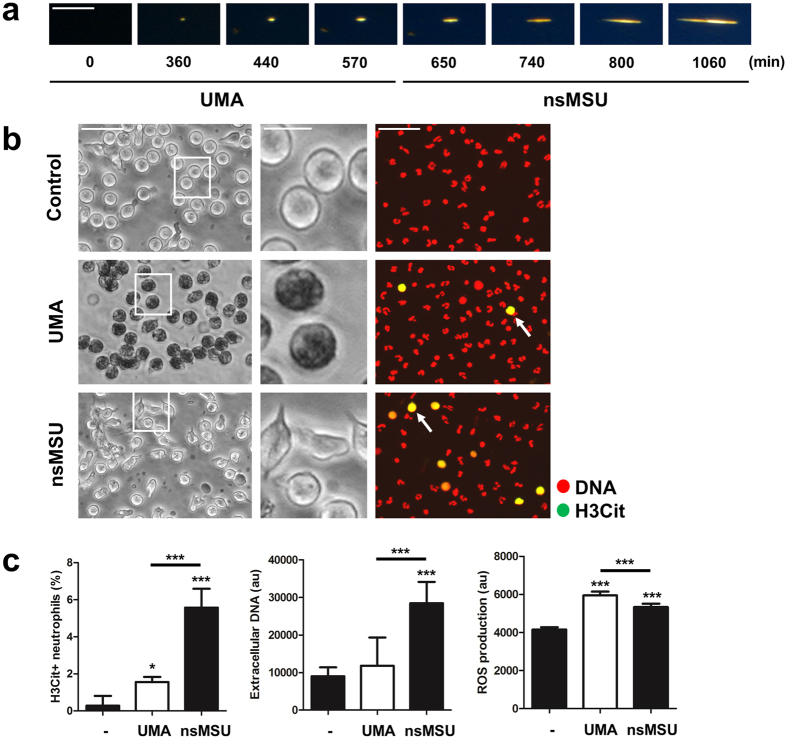
Impaired phagocytosis of UMA leads to the formation of nsMSU with NET-inducing potential. (**a**) In the absence of phagocytes, nsMSU developed in hyperuricemic serum. (**b**) Ingestion by neutrophils of huge amounts of UMA and nsMSU (left panels) caused moderate and substantial hypercitrullination of histone H3 in cultured granulocytes (right panels, white arrows), respectively. (**c**) Citrullination of histone H3 (left panel) and release of DNA (middle panel) preferentially occurred in response to nsMSU, whereas ROS production was significantly higher in response to both UMA as well as nsMSU (right panel). Scale bars: 30 μm (inserts: 10 μm). ***p < 0.001, *p < 0.05.

We observed that UMA ultimately grow at their longitudinal ends into nsMSU (15–20 μm in size) in the absence of phagocytes (Fig. 4a; Movie S2). The growth of UMA into nsMSU also occurred in the presence of phagocytes and heat-inactivated hyperuricemic serum, thus in a condition in which the clearance of UMA by phagocytes is impaired due to the lack of complement proteins (Supplementary Figure 3). In contrast, nsMSU were not observed in the presence of phagocytes and hyperuricemic serum that was not heat-inactivated. Collectively, these data indicate that the impaired clearance of UMA by blood-borne phagocytes can promote the development of nsMSU in hyperuricemic serum. Since neutrophils can sense microbe sizes and selectively release NETs toward large pathogens^23^, we assessed whether the size of crystals (UMA versus nsMSU) influences NET release. For this purpose, we incubated purified neutrophils for 4 hours separately with UMA (≤1 μm) or nsMSU (15–20 μm) and analyzed ROS production, extracellular DNA release and the presence of citrullinated histone H3 as markers of NETosis. Neutrophils stimulated with UMA had a highly granular phenotype, indicative for the uptake of these crystals, but barely stained positive for citrullinated histone H3 (Fig. 4b,c, left panel). In line with this, no extracellular DNA was detected when neutrophils were incubated with UMA (Fig. 4c, middle panel). In contrast, nsMSU promoted substantial citrullination of histone H3 and induced robust DNA release by neutrophils. Intriguingly, ROS production was increased in response to both UMA and nsMSU with a reciprocal difference in favor of UMA (Fig. 4c, right panel). Taken together, these data indicate that neutrophils can distinguish small from big crystals and selectively release NETs in response to the longer nsMSU, which can occur when UMA are insufficiently cleared.

### Intravenously injected UMA end up in the spleen and liver

**Figure 5:**
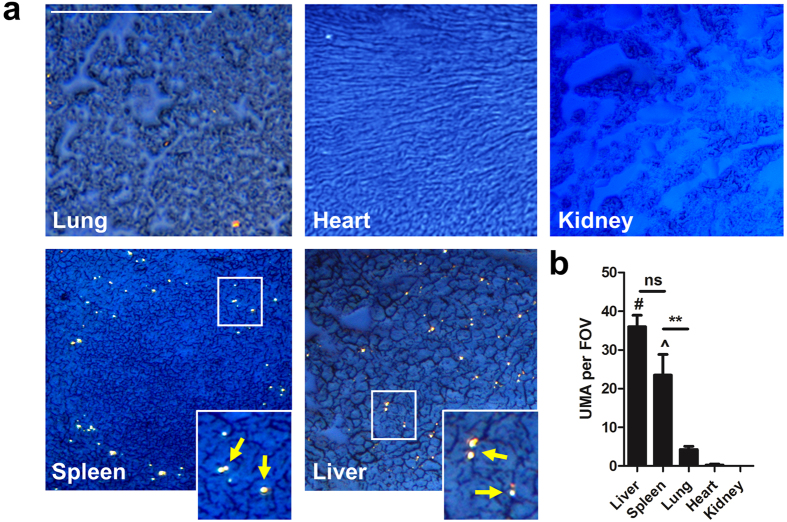
Circulating UMA undergo splenic and hepatic clearance *in vivo*. (**a**) Intravenously injected UMA (1 mg) are cleared in mice in the sinusoids of the liver or in the marginal zone of the spleen (yellow arrows indicate UMA), whereas other organs contained no (heart, kidneys) to very little (lungs) UMA. (**b**) The amount of UMA per organ were quantified by analyzing at least four different fields of view (FOV) at 200x magnification for the presence of UMA. Scale bar: 100 μm. **p < 0.01, ^#^p < 0.001 compared to lung, heart and kidney, ^p < 0.001 compared to heart and kidney.

When we injected 1 mg UMA intravenously in the tail vein of Balb/c mice, we observed the crystals ended up within the sinusoids of the liver and in the marginal zones of the spleen, whereas the heart and kidneys contained no UMA (Fig. 5a). Blood smears from these mice did not contain UMA either (not shown). Only a few UMA were observed within the lungs (Fig. 5b), which appeared a bit larger in size than regular UMA. The observation that the lungs, which are the first organs to pass after injecting UMA in the tail vein, contained very little UMA implies that UMA are cleared from the circulation by specific organs, i.e. the spleen and the liver. Experiments with nsMSU could not be performed *in vivo* since these crystals caused sudden death in pilot experiments. Therefore, we studied nsMSU under condition of flow *in vitro*.

### Under venous flow, UMA and nsMSU both induce NETosis

**Figure 6:**
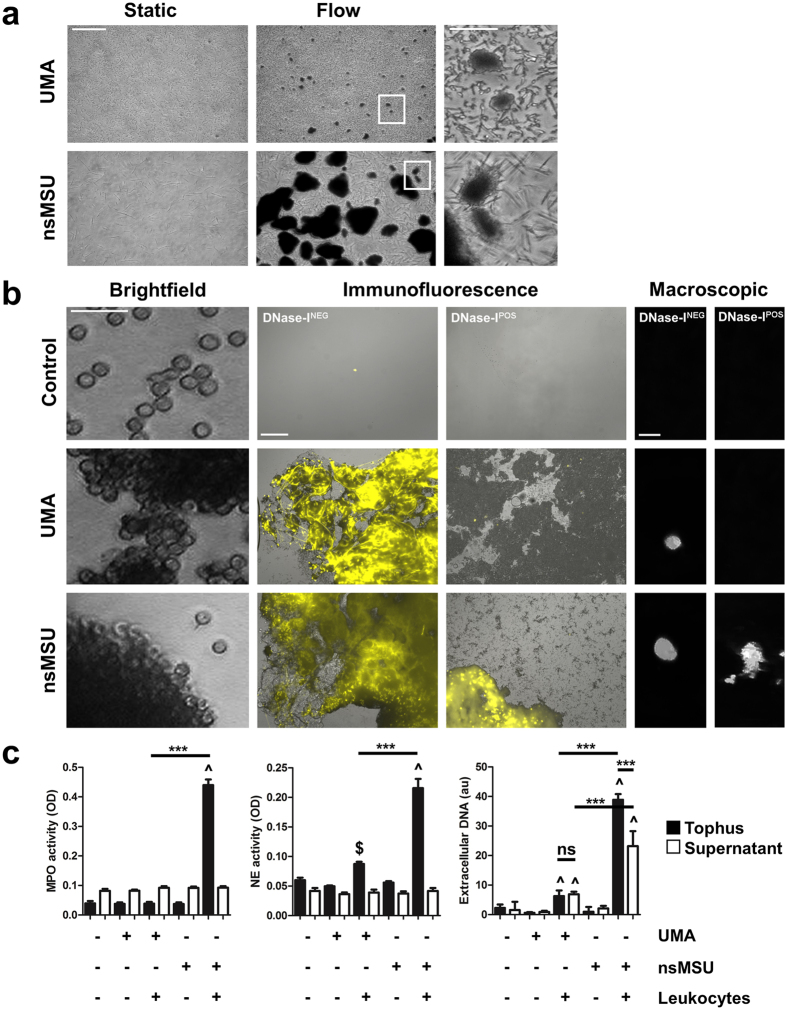
cMSU-induced NETosis promotes tophus formation. (**a**) Under conditions of flow, high concentrations of UMA and nsMSU formed large aggregates of crystals. (**b**) Leukocytes were attracted to UMA and nsMSU aggregates (left panels) and released NETs (DNase-I^NEG^; middle panels), thereby forming macroscopic tophus-like structures (right panels). Aggregates of NETs and nsMSU resisted DNase I-mediated degradation (DNase-I^POS^; middle panels), whereas UMA-induced NETs were completely dissolved. (**c**) nsMSU-containing tophi were highly positive for active myeloperoxidase (MPO), neutrophil elastase (NE) and extracellular DNA. Scale bars: (**a**) 30 μm (inserts: 10 μm); (**b**) ‘brightfield’ 30 μm, ‘immunofluorescence’ 100 μm, ‘macroscopic’ 3 mm. ^p < 0.001 compared to control situation, ^$^p < 0.05 compared to control situation, ***p < 0.001 between indicated conditions.

Next, we repeated our assays under conditions simulating a venous flow rate. At high crystal concentrations used in earlier experiments, we observed that UMA and nsMSU formed tiny and massive crystal aggregates, respectively (Fig. 6a). This appeared to be a physical flow-dependent process occurring in the absence of leukocytes. However, the high concentration of UMA is a non-physiological condition, since our data suggest that UMA are unable to reach these concentrations under physiological conditions due to their rapid clearance by blood-borne phagocytes. The size of these aggregates drastically increased in the presence of leukocytes (Fig. 6b, left panels ‘Brightfield’) forming macroscopic tophus-like structures with a diameter of up to 3 mm (Fig. 6b, right panels ‘Macroscopic’). Further analyses revealed that these aggregates contained high amounts of extracellular DNA (Fig. 6b, middle panels ‘Immunofluorescence’) with nsMSU and UMA at high doses. In contrast to static culture conditions, high amounts of UMA aggregates appear capable of inducing NETosis under flow. This is presumably caused by physical aggregation of these crystals forming structures too large for immediate phagocytic clearance. The extracellular DNA appeared particularly densely packed in nsMSU aggregates. Importantly, DNA aggregates containing nsMSU resisted digestion by DNase I, most likely by stabilization of the aggregate by the large sized crystal framework, whereas UMA-containing DNA aggregates were sensitive to DNase I and got fully dissolved (Fig. 6b, middle panels ‘Immunofluorescence’). Notably, nsMSU-containing DNA aggregates contained much more myeloperoxidase activity, neutrophil elastase activity, and extracellular DNA than UMA-containing DNA aggregates (Fig. 6c). In sum, UMA and nsMSU induce NETosis under flow, by which large macroscopic aggregates of NETs and crystals are formed. However, the UMA-based aggregates are readily dismantled by DNase I.

### NETosis shields endothelial cells from pro-inflammatory UMA and nsMSU

**Figure 7:**
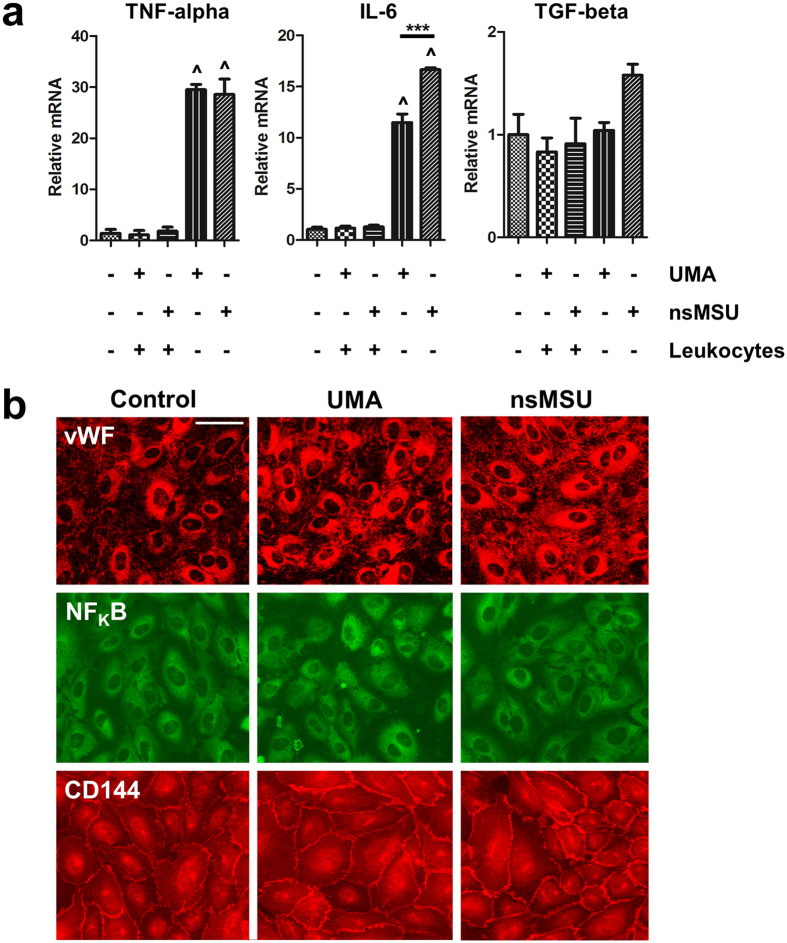
NETosis protects endothelial cells from the pro-inflammatory effects of cMSU. (**a**) UMA and nsMSU induce mRNA upregulation in HUVEC of tumor necrosis factor α (TNF-α) and interleukin 6 (IL-6), whereas mRNA expression of transforming growth factor β (TGF-β) remained constant. The upregulation of TNF-α and IL-6 mRNA in HUVEC was prevented by the presence of leukocytes (i.e. erythrocyte-depleted whole blood). (**b**) Immunofluorescence imaging of HUVEC shows that tophus-like structures of UMA or nsMSU and extracellular DNA does not promote release of von Willebrand factor (vWF; upper panels), nuclear translocation of NF_K_B p65 (middle panels) or loss of VE-cadherin (CD144)-mediated junctions (lower panels). Scale bar: 20 μm. ^p < 0.001 compared to control, ***p < 0.001 between indicated conditions, **p < 0.05 between indicated conditions.

Intravascular NETosis was previously linked to an altered vascular integrity during acute lung injury^13^. We therefore assessed the consequences of UMA- and nsMSU-induced NET formation on endothelial cells. For this purpose, we stimulated confluent HUVEC monolayers under flow for 6 hours with UMA or nsMSU. In the absence of leukocytes, both UMA and nsMSU induced an upregulation of TNF-α and IL-6 mRNA in HUVEC, whereas TGF-β mRNA levels remained constant (Fig. 7a). However, in the presence of leukocytes (i.e. erythrocyte-depleted whole blood), TNF-α and IL-6 mRNA levels in HUVEC were not upregulated compared to unstimulated HUVEC, indicating that leukocytes shield endothelial cells from the pro-inflammatory effects of cMSU. Notably, we did not observe cytotoxicity of these structures to endothelial cells despite the formation of large macroscopic aggregates in culture supernatants, which contained cytotoxic histones and active proteases. The endothelial cells and the integrity of the endothelial monolayer remained intact, as determined by immunofluorescence imaging of the adherens junction protein VE-cadherin (CD144) (Fig. 7b). In addition, we found no release of von Willebrand factor (vWF) or nuclear translocation of NF_K_B (Fig. 7b), indicating that endothelial cells are also not activated by these cMSU-containing aggregates. Interestingly, immunofluorescence imaging revealed that UMA-stimulated leukocytes adhered significantly more to endothelial cells than unstimulated or nsMSU-stimulated leukocytes (not shown). In summary, leukocytes protect endothelial cells from cMSU-induced damage.

## Discussion

The formation and growth of cMSU is a complex process that is influenced by multiple factors, including temperature, calcium levels, pH and/or mechanical shock^24^. Preferentially, nsMSU develop within the relatively cold synovial fluids or within the acidic urine, giving rise to gouty arthritis and renal calculi, respectively. Whether uric acid also crystallizes within hyperuricemic blood has long been a subject of debate. The observation that the solubility of uric acid increases in the presence of albumin has supported the notion that uric acid does not form crystals in blood^25^. However, the role of albumin in uric acid crystallization is controversial. Some investigators suggested that albumin strongly inhibits nsMSU formation, some claimed that albumin has absolutely no effect, while others reported that albumin accelerates the crystallization process^3^. Our data refute the notion that albumin inhibits uric acid crystallization. We found that small, round-shaped UMA developed within hyperuricemic serum as well as whole blood *ex vivo*. These UMA were taken up by blood-borne phagocytes and cleared from the circulation. They finally ended up in the sinusoids of liver and in the spleen.

The abundant plasma proteins fetuin (also known as alpha-2-HS-glycoprotein) and complement promoted the clearance of UMA by blood-borne phagocytes. Indeed, complement proteins can bind to and get activated by nsMSU^14^,^15^,^16^. A role for fetuin in the phagocytosis of crystallized uric acid has not been reported yet, although the contribution to the clearance of calprotein-like particles^26^, apoptotic cells^27^ and polystyrene nanospheres^28^ is well established. Interestingly, fetuin enhanced the phagocytosis of UMA only in the presence of active complement. Cooperative effects of fetuin and serum proteins were previously reported in the context of calcium phosphate crystal nidi^29^. UMA intravenously injected into mice were traced back in the sinusoids of the liver and the marginal zones of the spleen (Fig. 5), which are both areas known to harbor specialized macrophages classified as part of the reticuloendothelial system^30^,^31^,^32^,^33^. Resident tissue macrophages in the liver and spleen are discretely positioned and transcriptionally programmed for the encounter of pathogens or environmental challenges, thus serving as immune sentinels^34^. Our observations thus suggest that the clearance of UMA by blood-borne phagocytes ultimately results in their hepatic or splenic exit from the circulation, where specialized macrophages sense UMA-loaded phagocytes^35^. The phagocytosis by macrophages of UMA-loaded neutrophils may be facilitated through the observed loss of CD31 on neutrophils that contain UMA. It has been proposed that CD31 expression on neutrophils prevents the ingestion of neutrophils by macrophages through transmitting active ‘detachment’ signals, whereby the loss of CD31 thus facilitates the ingestion of neutrophils by macrophages^20^.

In hyperuricemic serum, in the absence of leukocytes, punctate UMA slowly formed and subsequently grew to nsMSU (Movie S2). The whole process is kinetically controlled and takes several hours. In contrast to UMA, single nsMSU triggered the release of potentially pro-coagulant NETs by blood-borne neutrophils in static culture conditions. Thus, an impaired clearance of UMA may bear the risk for thrombus formation and vascular injury. From previous work, we know that the release of NETs in response to nsMSU involves the generation of ROS^4^. We observed that both UMA and nsMSU induced ROS production in neutrophils, whereas citrullination of histone H3 and DNA release, two major hallmarks of NETosis, were particularly observed in response to nsMSU. This suggests that the inflammatory response of neutrophils towards crystallized uric acid is dependent on the size of the crystals. Indeed, neutrophils possess the ability to sense pathogen size^23^. The observation that high amounts of UMA, forming extended aggregates under conditions of flow, induced NETosis supports this line of thought.

In contrast to other work^36^,^37^, we did not observe cytotoxic effects of crystal-induced NETs to endothelial cells. Instead, the presence of leukocytes prevented the pro-inflammatory effects of UMA and nsMSU to endothelium. This process involved the trapping of the crystals by extended aggregated NETs. Thus, although it is generally accepted that cMSU act pro-inflammatory through ligation of toll like receptors and subsequent activation of NF_K_B signaling^38^,^39^,^40^, the pro-inflammatory effects of cMSU are reduced when these are entrapped within NETs. Of note, we found that DNase I only dismantled and dissolved UMA-containing aggregated NETs, whereas those composed of nsMSU were rather resistant to DNase I activity.

**Figure 8:**
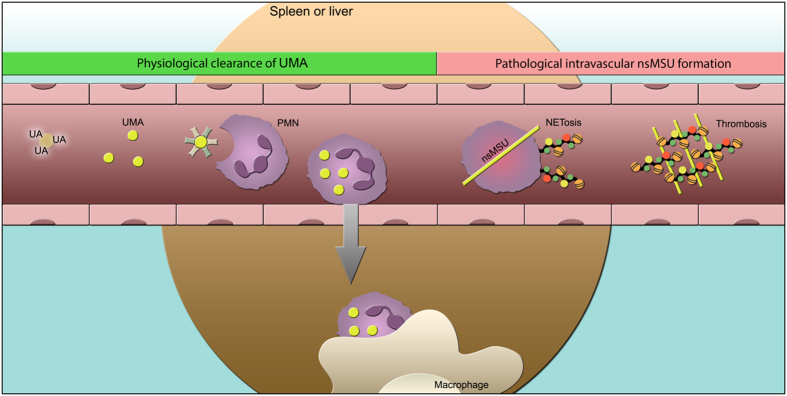
Hypothetical model for the mechanisms beyond and the relevance of UMA clearance from hyperuricemic blood. UMA are the first crystals that are to be observed in hyperuricemic blood after several hours of incubation. They are instantly taken up by blood-borne phagocytes before they grow to nsMSU. The phagocytosis is fostered by opsonization with complement and fetuin. The UMA-loaded phagocytes are consecutively cleared from circulation by resident macrophages in the marginal zone of the spleen or the sinusoids of the liver. If not properly cleared, UMA grow to hazardous NET-inducing nsMSU. This carries the risk of intravascular tophus- and thrombus-formation and obstruction of the vessel.

Altogether, we propose a model in which complement proteins and fetuins facilitate the cooperative clearance by blood-borne phagocytes and resident macrophages of UMA that spontaneously form in hyperuricemic blood (Fig. 8). The relevance of this clearance mechanism is reflected by the capacity of nsMSU to induce NETosis, which may have detrimental effects if it occurs in the vasculature. In otherwise healthy individuals with hyperuricemia, UMA will be cleared from the circulation in statu nascendi. This prevents a further crystal growth and the formation of potentially hazardous nsMSU. Since the clearance of UMA is significantly impaired in the absence of complement factors or fetuin, patients with chronic kidney disease and/or low levels of these plasma proteins^41^,^42^,^43^,^44^,^45^ may potentially be at risk to develop chronic NET-driven vascular inflammation.

## Materials and Methods

### Patient material

Serum and heparinized whole blood was collected from eight hyperuricemic patients and normouricemic controls and uric acid concentrations were determined by routine laboratory methods. All patient samples analyzed were anonymously donated to our research faculty and exceeded a uric acid concentration of 12 mg/dL. Some samples were found to be hypersaturated with uric acid up to 21 mg/dL, fairly above the solubility limit of approximately 7 mg/dL. Serum and whole blood was incubated at 4 °C and 37 °C and analyzed for the formation of crystalline structures employing polarized light video microscopy. Healthy donors served as controls. For some experiments, serum was collected from complement deficient patients (C4A, C4B, C4, C2 or C1q). Informed consent for blood donation was obtained from all subjects for all methods. All experiments with human material were performed in accordance with institutional guidelines and were approved by the Ethical committee of the University Hospital Erlangen.

### Animals

Balb/c mice were bred at the Danylo Halytsky Lviv National Medical University, Lviv, Ukraine, during Jan – August 2014 and kept on a standard diet with drinking water available ad libitum. All animal studies were performed in accordance with the guidelines determined by Law of Ministry of Healthcare of Ukraine, No. 281 from 01.11.2011 for the care and use of laboratory animals and approved by Ethics Council of the Danylo Halytsky Lviv National Medical University, protocol 1/6, dated June 24, 2013.

### Clearance of UMA *in vivo*

For *in vivo* experiments, 1 mg UMA or nsMSU were intravenously injected in 250 μl PBS into Balb/c mice. Cryosections of heart, kidneys, lungs, spleen and liver were prepared without any fixation 24 hours after the injection and analyzed by polarized light microscopy. Since animals immediately died after injection of nsMSU, we only used UMA for our final analyses for ethical reasons.

### Isolation of neutrophils

Neutrophils were isolated from heparinized (20 U/mL) blood by density gradient centrifugation using Lymphoprep (Stemcell Technologies). Briefly, whole blood was diluted 1:1 in PBS to a volume of 30 mL and slowly layered on top of 15 mL Lymphoprep. The blood was then centrifuged at 800 g for 20 minutes and neutrophils were collected from the high density fraction. Residual erythrocytes were eliminated by repetitive hypotonic lysis. Viable neutrophils were counted in a Neubauer cell counting chamber.

### Phagocytosis assays

For *ex vivo* phagocytosis experiments, anticoagulated fresh whole blood was incubated at 37 °C with UMA. Erythrocytes were subsequently removed by hypotonic lysis. Leukocytes were stained with anti-CD16 (cat. no. B-E16-FITC, Abcam) and anti-CD14 (cat. no. 61D3-PE, eBioscience) and analyzed by flow cytometry. For *in vitro* phagocytosis experiments, purified neutrophils (2 × 10^6^ cells/ml) were stimulated with 50 pg of UMA per cell in the presence of 10% autologous serum. Serum had been pre-treated as indicated. The phagocytic indices (PhIx) for UMA were calculated by multiplying the percentage of cells with increased side scatter with the mean side scatter value. Where indicated, phagocytosis was confirmed by exposing neutrophils to UMA in the presence of 100 nM pHrodo Red SE (Life Technologies) and subsequently analyzing cell-borne fluorescence by flow cytometry. Flow cytometry and data analyses were performed employing the Gallios cytofluorometer and the Kaluza software, respectively (Beckman Coulter).

### Neutrophil-macrophage co-cultures

Monocytes from healthy donors were isolated from peripheral blood mononuclear cells (PBMC) by plastic adherence for 2 hours in serum-free media. They (1 × 10^6^ cells/ml) were cultured for 7 days in RPMI1640 (Gibco) supplemented with 10% FCS and 25 ng/ml GM-CSF (Peprotech) to obtain monocyte-derived macrophages (MDMs). For neutrophil-macrophage co-culture experiments, purified neutrophils were stimulated with 50 pg of UMA per cell for 30 minutes and subsequently co-incubated with MDMs for 4 hours at a neutrophil:macrophage ratio of 4:1. At the indicated time points, neutrophils were harvested and analyzed by flow cytometry. Internalization of UMA-containing neutrophils (labeled with PKH26, Sigma-Aldrich) by MDMs was confirmed by immunofluorescence microscopy. Macrophages were visualized by staining the actin cytoskeleton using phalloidin-FITC (Sigma-Aldrich). Where indicated, neutrophils were pre-labeled with 30 nM pHrodo Red SE (for 30 min, followed by washing twice) prior to their use in co-culture experiments with macrophages. To check the status of neutrophils after UMA exposure, we stained neutrophils for phosphatidyl serine (Annexin V-FITC, cat. no. K101–100, Biovision; according to the manufacturer’s instructions), CD11a (cat. no. 559875, BD Biosciences), CD18 (cat. no. 555923, BD Biosciences) and CD31 (cat. no. MHCD3101, ThermoFisher) and analyzed the expression of these surface molecules by flow cytometry after 2 and 4 hours exposure to UMA.

### NETosis assays

Purified neutrophils were stimulated with 50 pg of crystals per cell (either UMA or needle-shaped nsMSU) for up to 4 hours, either under static conditions or under conditions of flow. For the latter, cells were either treated with crystals on a rocking table or within a pump system yielding a laminar flow rate of 20 ml/min (Ibidi). NETosis was assessed by quantifying DNA release with fluorometry or by immunofluorescence microscopy. For both techniques, extracellular DNA was stained with 100 nM Sytox Orange. The activity of NET-associated neutrophil elastase (NE) and myeloperoxidase (MPO) was determined by colorimetry, using 100 μM N-methoxysuccinyl-Ala-Ala-Pro-Val 4-nitroanilide (Sigma-Aldrich) or 1 mM 3′,5,5′-tetra-methylbenzidine (eBioscience) as substrates for NE and MPO, respectively. For the measurement of ROS, neutrophils were stimulated in the presence of 40 μM 2′,7′-dichlorofluorescin diacetate (Sigma-Aldrich).

### Endothelial cell culture and real-time PCR

Confluent monolayers of human umbilicial vein endothelial cells (HUVEC) were treated under flow with UMA or nsMSU, either in the presence or absence of purified leukocytes (i.e. erythrocyte-depleted whole blood). The viability and activation status of endothelial cells were analyzed by trypan blue exclusion assays, immunofluorescence microscopy or qRT-PCR. For immunofluorescence microscopy, HUVEC were stained for von Willebrand factor (Dako, A0082), NF_K_B p65 (Santa Cruz, sc-8008) and VE-cadherin (Santa Cruz, sc-6458). For qRT-PCR analyses, RNA was isolated from HUVEC with TRIzol© reagent (Ambion) and qRT-PCR analyses were performed on tumor necrosis factor alpha (TNF-α; forward 5′-CCCAGGCAGTCAGATCATCTTC-3′ and reverse 5′-GGCAGAGGTAGGTCTGGTTC-3′), interleukin 6 (IL-6; forward 5′-AGCCACTCACCTCTTCAGAAC-3′ and reverse 5′-CGGGACTCTTTCCTCTGTACA-3′) and transforming growth factor beta (TGF-β; forward 5′-GGATGGTGGAAGGTCTCATTTTA-3′ and reverse 5′-CGACTATGGTAGGGTTATCGACA-3′). GAPDH (forward 5′-GAAGGTGAAGGTCGGAGT-3′ and reverse 5′-CTTTAGGGTAGTGGTAGA-3′) was used as housekeeping gene. Fold change was calculated using the delta-delta Ct method.

### Production of nsMSU and UMA

A solution of 10 mM uric acid and 154 mM NaCl (pH 7.2) was agitated for 3 days to form nsMSU. The resultant crystals were washed with ethanol and sterilized at 180 °C for 2 hours prior to storage in PBS. For experiments with UMA we extensively grinded nsMSU under sterile conditions using Precellys ceramic (1.4 mm) beads. The similarity in size and shape between these artificially produced UMA and those UMA observed in hyperuricemic blood was assessed with polarized light microscopy.

### Statistical analyses

Results are represented as mean ± SEM of at least three and up to ten independent experiments. Significance was either determined by Student’s t-test or one-way ANOVA followed by Bonferroni correction using GraphPad Prism. P-values ≤ 0.05 were considered as statistically significant.

## Additional Information

**How to cite this article**: Pieterse, E. *et al*. Blood-borne phagocytes internalize urate microaggregates and prevent intravascular NETosis by urate crystals. *Sci. Rep.* **6**, 38229; doi: 10.1038/srep38229 (2016).

**Publisher's note:** Springer Nature remains neutral with regard to jurisdictional claims in published maps and institutional affiliations.

## Supplementary Material

Supplementary Data

Supplementary Movie 1

Supplementary Movie 2
