# Supplementary material for: Blood-borne phagocytes internalize urate microaggregates and prevent intravascular NETosis by urate crystals
Source: Sci Rep. 2016 Dec 5;6:38229. doi: 10.1038/srep38229 (PMC5137018; doi:10.1038/srep38229)

**Blood-borne phagocytes internalize urate microaggregates and prevent intravascular NETosis by urate crystals**

**Elmar Pieterse^1,2^, Ivica Jeremic^2,3^, Christine Czegley^2^, Daniela Weidner^2^, Mona H.C. Biermann^2^, Susan Veissi^2^, Christian Maueröder^2^, Christine Schauer^2^, Rostyslav Bilyy^2,4^, Tetiana Dumych^4^, Markus Hoffmann^2^, Luis E. Munoz^2^, Anders A. Bengtsson^5^, Georg Schett^2^, Johan van der Vlag^1*^ and Martin Herrmann^2*^**

^1^ Department of Nephrology, Radboud University Medical Center, Nijmegen, the Netherlands

^2^ Department of Internal Medicine 3, Friedrich-Alexander-University Erlangen-Nuremberg, Erlangen, Germany

^3^ Institute of Rheumatology, Resavska 69, Belgrade, Serbia

^4^ Danylo Halytsky Lviv National Medical University, Lviv, Ukraine

^5^ Department of Rheumatology, University Hospital of Lund, Lund, Sweden

*JV and MH equally contributed to senior authorship

**Keywords:** uric acid, monosodium urate, hyperuricemia, gout, neutrophil extracellular traps, clearance

**Correspondence to:** Luis E. Munoz, Department for Internal Medicine 3, Erlangen University Hospital, Friedrich-Alexander University of Erlangen-Nuremberg, Krankenhausstr. 12, 91054, Erlangen, Germany.

E-mail: [Luis.Munoz@uk-erlangen.de](mailto:Martin.Herrmann@uk-erlangen.de)

Johan van der Vlag, Department of Nephrology, Radboud University Medical Center, Geert Grooteplein Zuid 10, 6500HB, Nijmegen, The Netherlands.

E-mail: [Johan.vandervlag@radboudumc.nl](mailto:Johan.vandervlag@radboudumc.nl)

**Supplementary Movies**

**Movie S1. Phagocytosis of UMA by neutrophils.** Purified neutrophils (top right) were incubated with

UMA (bottom left) and filmed for 80 minutes at 37ºC by polarized light video microscopy.

**Movie S2. Formation of μMSU and subsequent growth to nsMSU.** The development of crystalline

structures in hyperuricemic serum was recorded by polarized light video microscopy for 18 hours at 37ºC.

**Supplementary Figures**

**Figure S1. Phagocytosis of UMA by neutrophils.** (A) Neutrophils dose-dependently increase in side scatter when exposed to UMA. (B) Neutrophils with an increased side scatter stain positive for pHrodo, indicating that those cells have truly internalized UMA. (C) To demonstrate a correlation between side scatter and the mean fluorescent intensity (MFI) of pHrodo, the population of pHrodo-positive neutrophils was divided into four equally-sized gates (left panel) and plotted for pHrodo MFI and side scatter (right panel). (D) The MFI of pHrodo correlates to the phagocytic index (PhIx) as calculated in our assays. (E) The percentage of UMA-containing neutrophils (i.e. pHrodo-positive neutrophils with increased side scatter) drops from 44% to 14% when serum is heat-inactivated (30 min, 55ºC) or pretreated with 500 µM EDTA to remove divalent cations.

**Figure S2. UMA-positive neutrophils remain viable but loose CD31 expression.** Unstimulated neutrophils or neutrophils exposed to UMA for 2 or 4 hours were stained for phosphatidyl serine (Annexin V staining) (A), CD31 (B), CD11a (C) and CD18 (D) and analyzed by flow cytometry. UMA-positive neutrophils were gated based on an increased side scatter (SSc).

**Figure S3. Impaired UMA clearance yields NET-inducing nsMSU.** UMA were incubated in hyperuricemic serum (uric acid levels of 20 mg/dL) in the presence of PKH26-labeled neutrophils (red) and the extracellular DNA-binding dye 4',6-diamidino-2-phenylindole (DAPI; blue) for 8 hours. Hyperuricemic serum was either heat-inactivated or not. In hyperuricemic heat-inactivated serum (two right panels), UMA grew into nsMSU (black arrows) and triggered the release of NET-like extracellular DNA. Such nsMSU and extracellular DNA were not observed for hyperuricemic serum that had not been heat-inactivated (middle panel). Scale bar: 50 µm (insert: 25 µm).

**Supplementary Figure 1**

**
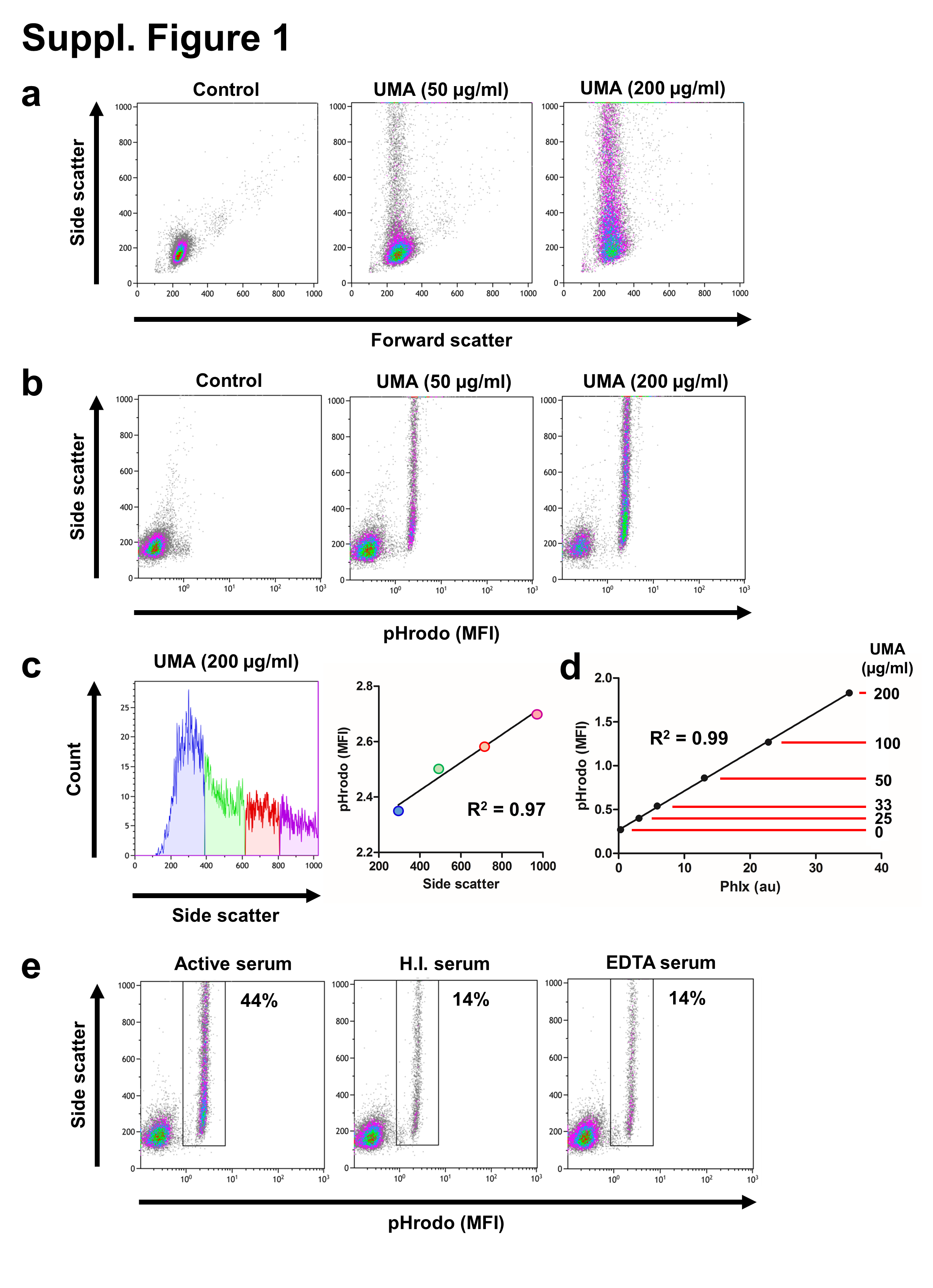
**

**Supplementary Figure 2**

**
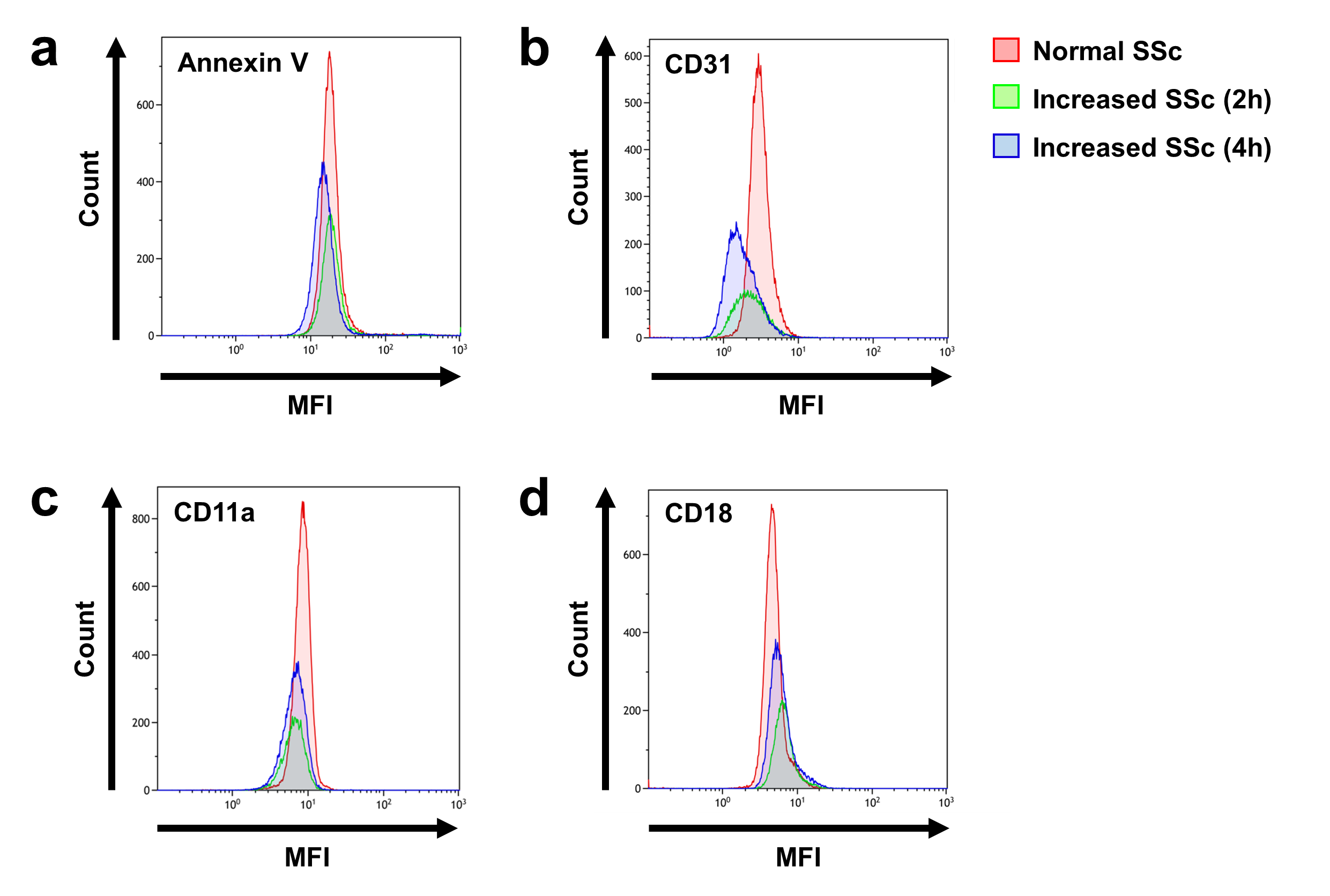
**

**Supplementary Figure 3**


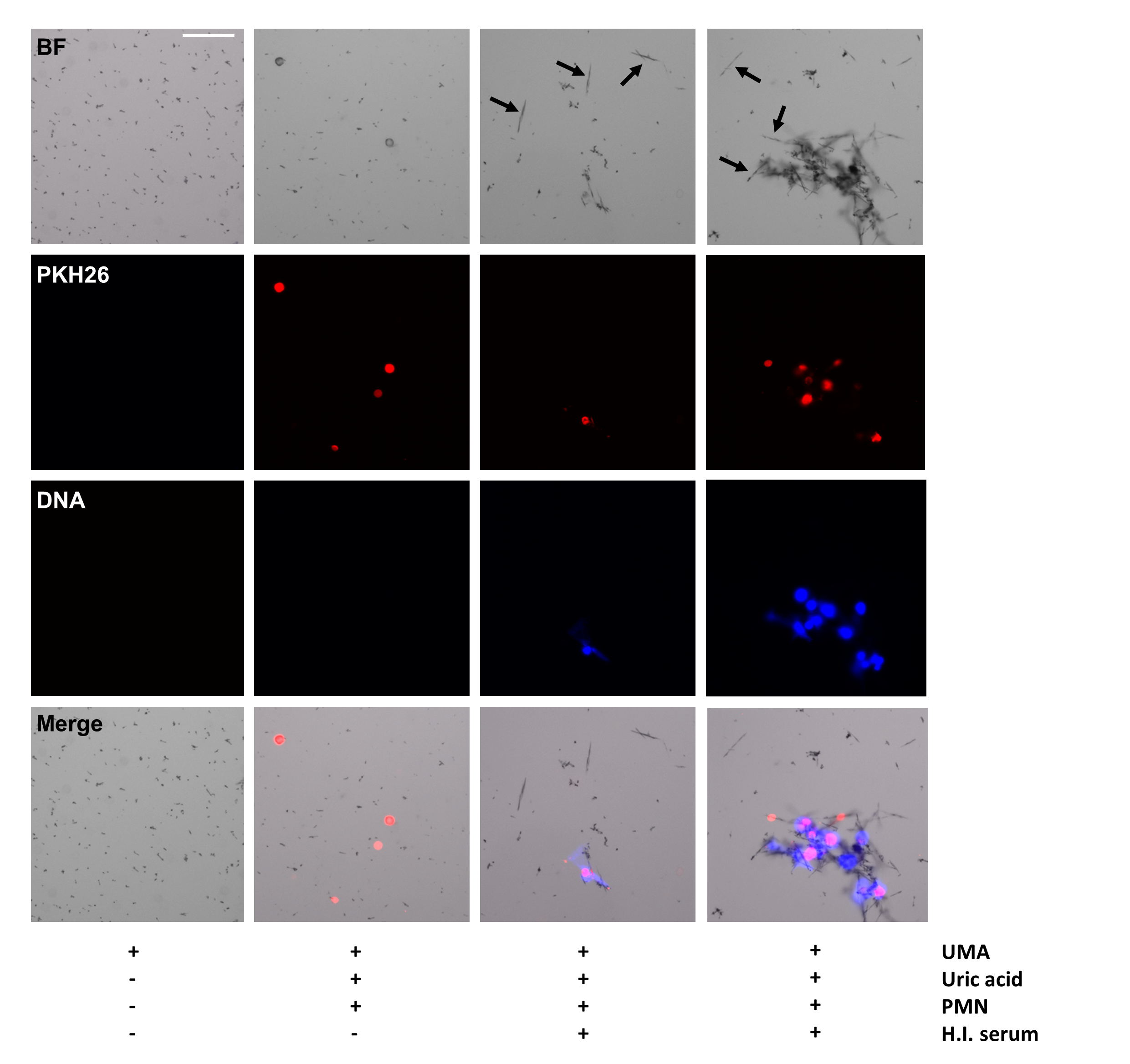

Supplement: Supplementary Data [file srep38229-s1.doc]
